# A Clinical Lecture upon Facial Neuralgia

**Published:** 1861-02

**Authors:** 


### MERCY HOSPITAL.

Service of E. ANDREWS, M. D., Prof. of Surgery.

### *A Clinical Lecture upon Facial Neuralgia*

GENTLEMEN:—The spouting blood and the gaping wounds of common surgery, do not in the least disturb me; yet I set a patient before you this morning, at whose horrible suffering, I confess I am appalled. This man presents to your view no perceptible organic disease. The heart and lungs are sound, the brain is normal in its condition, no canker gnaws at his vitals and no poisonous disease contaminates his blood; yet this man, for very torture, has not lain down in bed for five years. His hair is gray with suffering rather than with age, and the firmness of his step, the steadiness of his voice, the brightness of his eye, and the vigor of his resolution, are all broken and gone, from the extremity of his distress.

Such a malady, gentlemen, is Facial Nuralgia in its more aggravated form.

This disease manifests itself, of course, in the branches of the fifth pair of nerves, from which the face derives its sensibility. Allow me to refresh your mind a little upon the anatomical relations of this nerve. The fifth pair while yet within the skull, and just at the body of the sphenoid bone forms the simi-lunar ganglion. From this ganglion proceed three large branches. The superior of ophthalmic branch supplies the eye, then runs along the roof of the orbit, passes through the supra-orbital foramen and supplies the forehead and adjacent parts. The middle or superior maxilary nerve runs straight forward through the foramen rotundum, across the spheno-maxilary fossa, into the orbit, whence it emerges by the infra-orbital foramen and supplies the cheek, upper lip, anterior part of the gum and the incisor teeth. The last or inferior maxillary branch of the fifth pair, passes out of the skull at the foramen ovale, and decends to the lower jaw where the chief branch enters the dental canal, supplies the lower teeth, and then emerging at the anterior mental foramen supplies the lower lip and the chin.

I show you here, a splendid French preparation in which all the branches and ganglion of the fifth pair are finely displayed with the various canals and foramina through which they pass.

To a careless practitioner, grown rusty in anatomy and obtuse in his pathology, facial neuralgia presents merely an obstinate darting pain in some part of the face, without any apparent inflammation, without much tenderness upon pressure and to his stupid apprehension without cause; but to the truly scientific surgeon, there is no more splendid opportunity for a clear diagnosis than one of these very cases.

To you, gentlemen, I trust that facial neuralgia will present no puzzles. When you have seen and examined your patient, the whole disease will be as clear to you as sunlight. You will know at once what nerve is affected, and will, without a moments hesitation, be able to put your finger upon the exact spot in its course where the cause of trouble exists; further more, you will be able to cure cases which other and less educated men have fumbled over in vain for years.

The key which unlocks all the mysteries of these cases is the following proposition:

*Facial Neuralgia, generally results from the compression of some of the branches of the fifth nerve, at the points of passage through the canals and foramina of the facial bones.*

If you question your patient, you will generally find that the pain centres chiefly in the parts supplied by a particular branch of the fifth nerve. If you trace back this nerve to the first foramen through which it passes, you will discover that all the branches on the distal side of the foramen are neuralgic, while all the branches given off before reaching the opening, and which do not pass through it, are unaffected. Thus for instance, this patient has a terrible shooting pain in the roots of the right canine and incisor teeth, in the outer side of the gum corresponding, in the upper lip overlying the affected teeth, and in the right cheek and the right lower eyelid. But he has no pain in the roof of the mouth, in the molar teeth, nor in the upper eyelid. Now then what nerves supply the front teeth, the outer side of the gum, the upper lip, the cheek, and the lower eyelid? Obviously the infraorbital nerve. Those twigs, and those only, are painful which have come through the infra-orbital foramen. This opening therefore must be the seat of the disease. To settle the question, I will now make pressure upon the foramen itself. (Here Prof. A. pressed upon the spot with the end of a pencil, and the patient cried out in a paroxysm of pain.) You see gentlemen, I have found the spot. Pressure at this foramen renews the atrocious pains, which seem to the patient to shoot through the whole distribution of the infra-orbital branches.

You remember the patient who was here some months since with an awful neuralgia in the lower lip and chin. His pain had endured seven years, and his life was utterly worthless to him. In that case, the pain was confined to those branches of the inferior maxillary nerve which passed through the mental foramen, and the excision of a portion of the trunk of the nerve on the proximal side of the foramen, effected a complete cure.

The pathology of these cases may be briefly elucidated as follows:

1st. The fifth pair of nerves above every other pair in the body sends its branches through a multitude of bony foramina.

2d. Any irritation of the bone or periosteum may cause constriction of the foramina or thickening of their fibrous lining, in which case the nerve is pressed upon and rendered neuralgic. Other tissues can accommodate themselves to a continuous pressure, so as not to be permanently troublesome, but it is a peculiarity of nerve trunks, that they will not thus submit, but keep up from year to year the most atrocious torments.

3d. From this it follows that constriction in the supra-orbital foramen will give rise to neuralgia of the forehead; in the infra-orbital foramen it will cause pain of the incisor teeth, upper lip and cheek; in the posterior palatine canal it will cause pain in the roof of the mouth where the palatine nerve is distributed; in the dental canal of the lower jaw it will effect the lower lip, the chin, and all the teeth anterior to the point of constriction.

The treatment suitable for neuralgia of the fifth pair, is in the first place to remove the constitutional diathesis, upon which the local constriction depends, as for instance, the rheumatic, syphilitic, or malarious influences. If this does not suffice, but the pain continues intolerable and incurable, then you must resort to surgical interference. The surgical operations adapted to these cases are of two kinds. The first is the subcutaneous injection of narcotics over the seat of constriction, and the second is the extirpation of a portion of the trunk of the nerve.

The first operation is one of recent invention, and was introduced into this country from France. The object is to introduce the sulphate of morphine or atropine directly in contact with the effected spot in the trunk of of the nerve, and thus effectually narcotize it. I confess, gentlemen, I am unable to account for the success which attends this operation. Thoretically, I should expect that the relief thus obtained would be very transient, lasting only until the narcotic injection was absorbed and disposed of; but the facts in my experience, and in the experience of others, show that the cure is often permanent after a few repetitions of the injection.

The means required for this operation are a proper syringe and a solution of narcotic. The syringe which I here show holds fifteen drops. The pipe is a fine canula of the size of a large needle, and sharp at the extremity for introduction into the tissue. The solution must be prepared of such strength that the syringe will hold the required dose of the narcotic, which must be the same as when given in full dose by the stomach.

The next step is to find the proper place of introduction. To do this you will bear in mind, that it is not to be thrown in at the place where the patient refers the pain, but over the foramen which is the seat of stricture. You will trace up the nerve therefore to its foramen, and there you will find a spot which being sharply pressed upon by any small hard object causes an aggravation of the neuralgic pain. This is the place for operation. In the present instance it is the infra-orbital foramen. I now place the point of the syringe over the foramen, push it through the skin, and inject the solution in direct contact with the nerve.

The effect of the injection is often surprising. I can refer to patients in whom a single injection has effected a cure that has now lasted for months, and will probably be permanent.

Sometimes, however, this method fails. The foramina which transmit the nerves are not all accessible to the syringe, and at other times it fails of its full success. In these cases you must resort to the extirpation of a portion of the nerve itself. In selecting the place, and mode of operation, you will be guided by the following rules:

1st. The portion of the nerve excised must be taken from the proximal side of the constricting foramen, and not merely from the proximal side of the seat of pain, otherwise the operation is a perfect failure, because the point where the nerve is pinched is still in communication with the brain.

2d. The portion of the nerve excised, must if possible exceed a half an inch in length. If it does not, there is danger that the nerve may be reproduced across the space, and the pain at a future time be renewed.

If the mental foramen of the inferior maxilla be the offending spot, you may operate at the angle of the jaw. Dissecting up the soft tissues, take a large trephine and place the centre pin upwards and forwards from the angle, at such a distance that the edge of the crown shall be within $\frac{1}{4}$ of an inch of the anterior edge of the ramus where it joins the body of the bone. Then cut through the external table and pry out the button with an elevator. The nerve will be found running across the bottom of the cavity and may be dessected out.

In the present instance the fault is at the infra-orbital foramen, In this case a flap must be raised from the cheek as high as the edge of the orbit. A trephine must then be applied to the front of the *autrum highmoranium* in such a manner that the edge of the crown shall cut close to the foramen. The button of bone being removed, the course of the nerve may be traced along the roof of the antrum by a slight bony ridge. This ridge is to be broken down with small forceps, or other instruments, and the nerve dissected out as far back as where it lies within the orbit, or, if deemed best even back to the spheno-maxillary fossa. The flap is to be laid down and secured with sutures.

If the supra-orbital nerve is in fault, no operation upon the bone is necessary. The nerve may be traced through the notch and cut out as far back in the orbit as you can reach without endangering the eye.

With these principles in your minds, gentlemen, you will find obstinate facial neuralgias not to be such incomprehensible riddles to you as they have been to your predecessors. You will understand them readily, and you will treat them efficiently, and the bewildered fumbling of former practice will so far as you are concerned be forever at an end.

